# The Nocardial *aph(2″)* Gene Confers Tobramycin and Gentamicin Resistance and Is an Effective Positive Selection Marker in Mycobacteria and Nocardia

**DOI:** 10.3390/microorganisms11071697

**Published:** 2023-06-29

**Authors:** Yizhak Hershko, Amos Adler, Daniel Barkan

**Affiliations:** 1 Koret School of Veterinary Medicine, The Robert H. Smith Faculty of Agriculture, Food and Environment, The Hebrew University of Jerusalem, Rehovot 7610001, Israel; itzikhershko@gmail.com; 2 Tel Aviv Sourasky Medical Center, and Tel Aviv University Faculty of Medicine, Tel Aviv 69978, Israel; amosa@tlvmc.gov.il

**Keywords:** 2″-O-phophotransferase *aph(2″)*, gentamicin, tobramycin, *Mycobacterium abscessus*, *Mycobacterium smegmatis*, *Mycobacterium tuberculosis*, *Nocardia asteroides*, selection marker

## Abstract

The current study aimed to evaluate the feasibility of using the aminoglycoside 2″-O-phosphotransferase *aph(2″)* gene as a positive selection marker in *N. asteroides*, *M. smegmatis*, *M. abscessus* and *M. tuberculosis*. The *aph(2″)* gene, known to confer resistance to tobramycin, was PCR amplified from *N. farcinica* and cloned into two plasmid vectors, pMSG383 and pDB151, harboring hygromycin and zeocin selection markers, respectively. The recombinant plasmids were transformed into the target microorganisms, and selectability was assessed against varying concentrations of tobramycin and using an E-test against gentamicin. The results indicated that the *aph(2″)* gene is a useful selection marker in Mycobacteria and Nocardia against tobramycin, with a good selectability at 2.5–10 µg/mL for *M. smegmatis* mc^2^-155 and *N. asteroides* ATCC 19,247, and 60–160 µg/mL for *M. abscessus* ATCC 19,977 and *M. tuberculosis* H37Ra. The minimum inhibitory concentration (MIC) of gentamicin for recombinant *N. asteroides*, *M. smegmatis* and *M. abscessus* was >256 µg/mL, whereas respective MIC in wild-type strains was 0.125 µg/mL, 0.38 µg/mL and 8 µg/mL, respectively. These findings demonstrate the potential of *aph(2″)* as a positive selection marker for genetic manipulation processes in Mycobacteria and Nocardia, thus facilitating their research and improving the efficiency of biotechnology applications. Conclusions: the *aph(2″)* gene is a useful, new selection marker for genetic manipulation of Nocardia and various Mycobacteria.

## 1. Introduction

The development of genetic manipulation techniques has been instrumental in advancing our understanding of microbial physiology, pathogenesis and metabolic capabilities, paving the way for novel biotechnological applications [1]. However, successful genetic manipulation requires the employment of reliable and efficient selection markers to facilitate the identification and maintenance of genetically modified organisms. Aminoglycoside antibiotics, which act by inhibiting protein synthesis in bacteria, are commonly used as selection agents in genetic engineering.

Mycobacteria and Nocardia are both actinobacteria that share several genetic and phenotypic characteristics, including a high guanine–cytosine (GC) content, complex cell wall structure and ability to produce a diverse range of bioactive compounds [2]. Some species within these genera, such as *N. asteroides*, *M. tuberculosis* and *M. abscessus*, are significant human pathogens [3,4], while others, like *M. smegmatis*, are utilized as model organisms in research [5]. The development of an efficient selection marker for these organisms could improve our understanding of their biology and facilitate their research.

The *aph(2″*) gene in *N. farcinica* coding, for an aminoglycoside 2″-O-phophotransferase, is known to confer resistance to tobramycin, an aminoglycoside antibiotic, by inactivating the drug through phosphorylation [6]. Previous studies have demonstrated the utility of other aminoglycoside resistance genes as selection markers, such as the neomycin [5] resistance gene. However, the potential of the *aph(2″)* gene as a positive selection marker in Mycobacteria and Nocardia has not been extensively explored. We hypothesized that this protein, coded by *N. farcinica*, can induce laboratory-significant tobramycin resistance in closely related bacteria such as *M. smegmatis*, *M. abscessus* and *M. tuberculosis* [7,8,9].

We therefore aimed to examine the feasibility of using the *aph(2″)* gene as a positive selection marker in *N. asteroides* ATCC 19247, *M. smegmatis* mc^2^-155, *M. abscessus* ATCC 19,977 and *M. tuberculosis* H37Ra. By cloning the *aph(2″)* gene into plasmid vectors and transforming them into target microorganisms, we evaluated its effectiveness and stringency as a selection marker in these organisms.

## 2. Materials and Methods

### 2.1. Bacterial Strains and Plasmids

Nocardia were grown in either BHI or LB liquid media, and LB or blood–agar solid plates. Mycobacteria were grown in 7H9 liquid media, supplemented with glycerol and tween, as previously widely described. *M. tuberculosis* media were also supplemented with oADC 10%. For solid plates, 7H10 media were used with the same additives except Tween.

### 2.2. Cloning and Expression of the aph(2″) Gene

We PCR-amplified the *aph(2″)* from *N. farcinica* NCTC 11,134 gDNA using the following primers: forward, 5′-AGATCTTTAAATCTAGATATCCATGATGGTCCGTCTCCCGCTCACGCCCG-3′; reverse, 5′-CATAAAGTGTCAAGCCTGGGGTCTGCATGAACCGACCAGACGCTACGGTT-3′. The 1.5 kb product, including the gene and the 300 bp preceding it, was cloned with a Gibson-like reaction into the BamHI site in pMSG383, an hygromycin-selected Mycobacterial/Nocardia/*E. coli* shuttle vector, creating pDB464. To clone the gene into a zeocin-selected shuttle vector, also coding for the red fluorophore mCherry, the gene was again PCR-amplified from pDB464 using the primers aph2-F-ecoRI-151 5′-GGCATGGACGAGCTGTACAAGTAAGTACCAGATCTTTAAATCTAGATATC-3′ and aph2-R-spe-151 5′-CTACGGGGTCTGACGCTCAGTGGAAGTTTCCGCGACCGATCGGCAACGGA-3′, and cloned using a Gibson reaction into the EcoRI and SpeI sites in pDB151, creating pDB474. pDB464 and pDB474 were introduced into target Nocardia and Mycobacteria with electroporation (2.5 kV, 1000 Ω, 25 µF, 2 mm cuvettes).

### 2.3. Antimicrobial Agents and MIC Determinations

Minimal inhibitory concentration (MIC) values were determined with Broth Micro Dilution (BMD) using the commercial Sensititre Rapmyco microdilution panel, according to the Clinical and Laboratory Standards Institute (CLSI) M24-A2 guidelines (CLSI, 2018) and the manufacturer′s instructions. Accordingly, isolates were subcultured on sheep blood agar for 24–48 h at 35 °C under aerobic conditions to ensure adequate growth and purity prior to antimicrobial susceptibility testing. Afterward, mature colonies were transferred to 1 mL of sterile water containing 3 mm sterile glass beads and were vortexed to obtain a homogenous suspension. Clumps were allowed to settle, and the supernatant was adjusted to a 0.5 McFarland standard using a nephelometer. Then, 50 µL of suspension was transferred to a Sensititre cation-adjusted Mueller–Hinton broth with a TES buffer (TREK Diagnostic Systems Ltd. East Grinstead, England). Each well of the panel was then inoculated with 100 µL of suspension and the panels were incubated at 35 °C for 48–72 h until moderate growth was observed in the positive control well. *N. asteroides* and *N. farcinica* NCTC 11,134 were used as the quality control strains. For the E-test, a gentamicin E-test strip (BioMérieux; https://www.biomerieux-usa.com/clinical/etest) was used on blood–agar plates.

### 2.4. Positive Selection Efficiency Experiments

In order to assess the gene’s selectivity for the specified bacterial strains (WT and the corresponding mutant strain), the target strains were inoculated on appropriate agar plates containing escalating concentrations of tobramycin. Bacterial cultures were then inoculated onto the plates from a 0.5 McFarland culture, using a 10 µL inoculation loop. Following this, the plates were incubated at 37 °C for 5 days for all bacteria, except *M. tuberculosis*, where the incubation was 25 days.

## 3. Results

### 3.1. The aph(2″) Gene Confers Resistance to Tobramycin and Gentamicin in Nocardia and Mycobacteria

**Figure 1:**
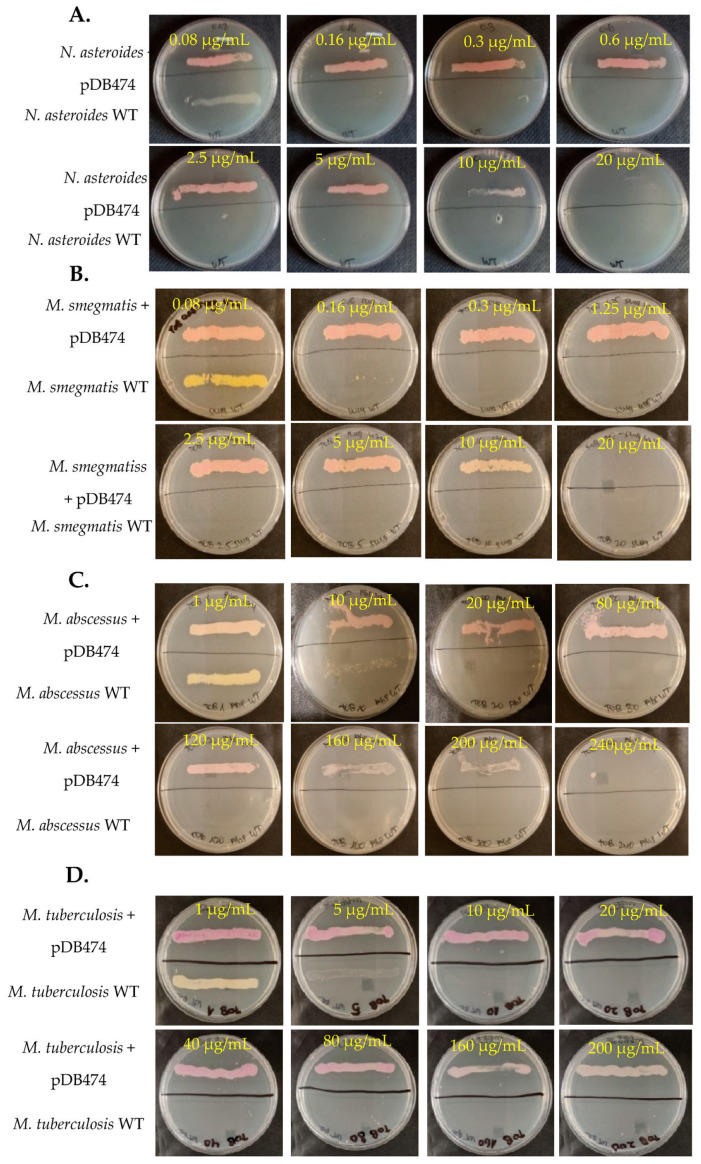
*N. asteroides* (**A**), *M. smegmatis* (**B**), *M. abscessus* (**C**) and *M. tuberculosis* (**D**), with the pDB474 plasmid (*aph(2″)* and mCherry) or WT, were streaked on the upper or lower sides of each plate, respectively. The concentration of tobramycin on each plate is designated. The Dishes are 9 cm diameter Petri Dishes.

**Figure 2:**
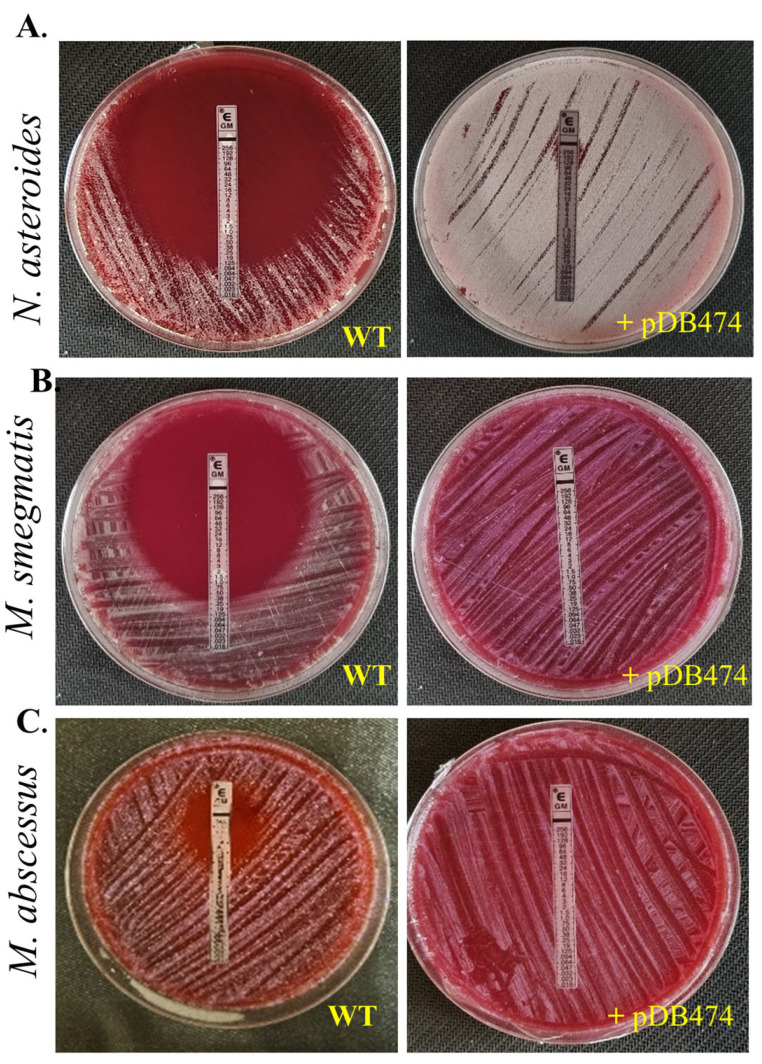
Gentamicin sensitivity was tested in *N. asteroides* (**A**)*, M. smegmatis* (**B**) and *M. abscessus* (**C**) by an E-test, in WT bacteria versus mutants harboring the pDB474 plasmid, carrying the *aph(2″)* gene from *N. farcinica.* Plates are 9 cm Petri dishes.

The *aph(2″)* gene was PCR-amplified from tobramycin-resistant *N. farcinica* NCTC 11,134 (MIC to tobramycin of 16 µg/mL) and cloned into the BamHI site in pMSG383 (a Mycobacterial/Nocardial/*E. coli* shuttle vector, with hygromycin selection), creating pDB464. This plasmid was then introduced into *N. asteroides* ATCC 19247, a tobramycin-susceptible strain lacking the gene. The MICs to tobramycin of the wild-type (WT), mutant and WT plus pMSG383 (empty vector control) strains were assessed using BMD. The mutant strain exhibited an MIC to tobramycin of 16 µg/mL, while the WT and WT plus pMSG383 strains had an MIC of 0.25 µg/mL (Table 1). To further characterize the phenotype conferred by this gene in both Nocardia and various Mycobacteria, we sub-cloned it into the Nocardial/Mycobacterial/*E. coli* shuttle vector pDB151, which also contains zeocin resistance and the red fluorophore mCherry, to create pDB474. pDB474 was electroporated into *N. asteroides*, *M. smegmatis*, *M. abscessus* and *M. tuberculosis* and plated on agar plates with the appropriate concentration of zeocin. After the typical incubation time, a zeocin-resistant, red-colored colony of each bacteria was picked and used for further testing. The MIC to tobramycin conferred by pDB474 in each of these bacteria was tested on agar plates with increasing concentrations of tobramycin, with the mutant (red-colored) and the WT bacteria both streaked on the same plate (Figure 1). As seen, in all the tested bacteria, pDB474 conferred a substantial increase in the MIC to tobramycin. The recombinant strains also demonstrated a substantially increased MIC to gentamicin (Figure 2).

### 3.2. The aph(2″) Gene Enables Effective Positive Selection for Plasmid Insertion in Nocardia and Mycobacteria

**Figure 3:**
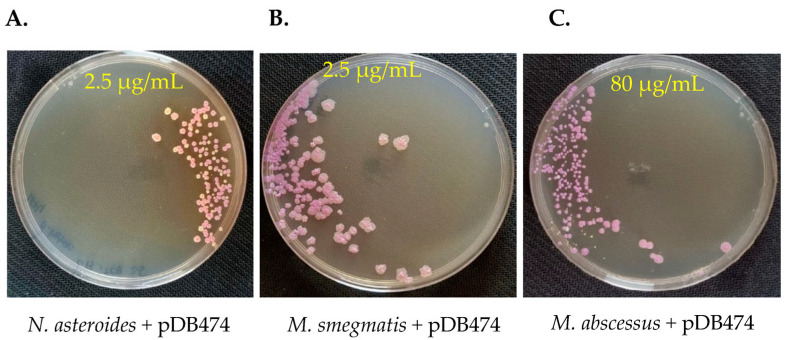
pDB474, a plasmid carrying mCherry and the *aph(2″)* gene, was electroporated into *N. asteroides* (**A**), *M. smegmatis* (**B**) and *M. abscessus* (**C**), and plated on the designated concentration of tobramycin. Correct transformants are of a reddish color, whereas background colonies are cream–white. Plates are 9 cm Petri dishes.

To examine whether the *aph(2″)* gene provides a selection stringent enough to be used as a positive selection marker in genetic manipulation of Nocardia and Mycobacteria, we again electroporated pDB474 into WT *N. asteroides, M. smegmatis* and *M. abscessus*, but this time plated them on agar plates with tobramycin. The concentration of tobramycin used for selection in each species was guided using the MIC results shown in Figure 1. As seen in Figure 3A, the vast majority of colonies obtained in *N. asteroides* are red, indicating they are indeed correct transformants. Still, a measurable proportion of colonies (~25%) appear to be background colonies. This proportion did not change significantly when the tobramycin concentration was increased to 5 µg/mL, and at 10 µg/mL, there was a substantial reduction in the recovery of colonies. We therefore conclude that although good-efficiency plasmid selection can be achieved using tobramycin and the *aph(2″)* gene, subsequent verification of plasmid insertion with a PCR (or other means) is warranted. In *M. smegmatis*, tobramycin of 2.5 µg/mL conferred excellent selection, with no apparent white colonies (Figure 3B). In *M. abscessus*, 80 µg/mL conferred very good selection with only a few white colonies, of which all but one were substantially smaller than the red ones (Figure 3C). We therefore conclude that the combination of tobramycin with the *aph(2″)* gene can effectively be used for positive selection, although some caution should be used, especially in Nocardia. This is especially important in *M. abscessus*, where positive selection markers are few and far between, and background colonies appear in all of them.

## 4. Discussion

In this study, we demonstrated the potential of the aminoglycoside 2″-O-phosphotransferase *aph(2″)* gene as a positive selection marker for genetic manipulation in Mycobacteria and Nocardia. The *aph(2″)* gene, which confers resistance to tobramycin, was successfully cloned into two plasmid vectors and transformed into *M. smegmatis*, *M. abscessus*, *M. tuberculosis* and *N. asteroides*. Our results revealed that the recombinant strains exhibited increased MICs to both tobramycin and gentamicin, indicating that the *aph(2″)* gene can serve as an effective selection marker in these organisms. 

Selectability of the transformed strains was found to be in the range of 2.5–10 µg/mL for *M. smegmatis* and *N. asteroides*, and 60–160 µg/mL for *M. abscessus* and *M. tuberculosis*. These findings highlight the broad applicability of the *aph(2″)* gene as a selection marker in diverse species within Mycobacteria genera. Importantly, our experimental findings provide direct evidence for the role of the *aph(2″)* gene in conferring resistance to gentamicin. While a previous study [6] proposed the *aph(2″)* gene as a potential explanation for the gentamicin-resistant phenotype, this current study is the first to experimentally demonstrate this phenomenon in Mycobacteria and Nocardia. Also, note that this gene is different from the *aph(3″)* gene previously described in Nocardia and Mycobacteria, conferring resistance to streptomycin [10] and kanamycin A [11].

Our study contributes to the ongoing efforts in developing and optimizing genetic manipulation techniques for Mycobacteria and Nocardia, which are crucial in furthering our understanding of their biology, pathogenesis and potential biotechnological applications. The use of efficient selection markers, such as the *aph(2″)* gene, could significantly enhance the success rate of genetic engineering in these organisms. Future studies may focus on investigating the applicability of the *aph(2″)* gene as a selection marker in other actinobacterial species, as well as optimizing transformation and selection conditions to further facilitate and improve the efficiency of genetic manipulation in these organisms.

## Figures and Tables

**Table 1:** MIC to tobramycin, as determined with Broth Micro Dilution, of *N. farcinica*, *N. asteroides*, recombinant *N. asteroides* + pMSG383 (harboring the hygromycin resistance gene) and *N. asteroides* + pDB464 [harboring both *hyg*^R^ and *aph(2″)*.

Strain/Plasmid/(Mutant Name)	MICs (μg/mL)
*N. farcinica* NCTC 11,134	16
*N. asteroides* ATCC 19,247 (wt)	0.25
*N. asteroides* ATCC 19,247 + pMSG383 (empty vector control)	0.25
*N. asteroides* ATCC 19,247/+ pDB464 [pMSG383 + *aph(2″)*]	16

## Data Availability

All data is available from the corresponding author upon request.
